# Incongruence between Verbal and Non-Verbal Information Enhances the Late Positive Potential

**DOI:** 10.1371/journal.pone.0164633

**Published:** 2016-10-13

**Authors:** Shu Morioka, Michihiro Osumi, Mayu Shiotani, Satoshi Nobusako, Hiroshi Maeoka, Yohei Okada, Makoto Hiyamizu, Atsushi Matsuo

**Affiliations:** 1 Neurorehabilitation Research Center, Kio University, 4-2-2 Umaminaka, Koryo, Kitakatsuragi-gun, Nara, 635-0832, Japan; 2 Department of Rehabilitation, Higashisumiyoshi Morimoto Hospital, 3-2-66 Takaai, Higashisumiyoshi, Osaka-city, Osaka, 546-0014, Japan; University of Toyama, JAPAN

## Abstract

Smooth social communication consists of both verbal and non-verbal information. However, when presented with incongruence between verbal information and nonverbal information, the relationship between an individual judging trustworthiness in those who present the verbal-nonverbal incongruence and the brain activities observed during judgment for trustworthiness are not clear. In the present study, we attempted to identify the impact of incongruencies between verbal information and facial expression on the value of trustworthiness and brain activity using event-related potentials (ERP). Combinations of verbal information [positive/negative] and facial expressions [smile/angry] expressions were presented randomly on a computer screen to 17 healthy volunteers. The value of trustworthiness of the presented facial expression was evaluated by the amount of donation offered by the observer to the person depicted on the computer screen. In addition, the time required to judge the value of trustworthiness was recorded for each trial. Using electroencephalography, ERP were obtained by averaging the wave patterns recorded while the participants judged the value of trustworthiness. The amount of donation offered was significantly lower when the verbal information and facial expression were incongruent, particularly for [negative × smile]. The amplitude of the early posterior negativity (EPN) at the temporal lobe showed no significant difference between all conditions. However, the amplitude of the late positive potential (LPP) at the parietal electrodes for the incongruent condition [negative × smile] was higher than that for the congruent condition [positive × smile]. These results suggest that the LPP amplitude observed from the parietal cortex is involved in the processing of incongruence between verbal information and facial expression.

## Introduction

When an individual experiences a particular emotion, it is usually manifest as a particular facial expression. For that reason, individuals infer other people’s emotions by observing their facial expressions. Recognizing facial expressions is an essential element in smooth human communication.

Many studies have examined brain activities related to the face and the recognition of facial expression. Using event-related potentials (ERP), Clark et al. analyzed facial recognition temporally [1]. Their results demonstrated that, when compared to the blank conditions used as a baseline, face stimuli activated the occipito-temporal lobe, including the fusiform facial area (FFA). In ERP, when facial images are displayed, a latency of approximately 170 ms negative (N170) is observed in the occipito-temporal lobe in the vicinity of the FFA [2].

Facial recognition is an important source of information for estimating the social characteristics of a target individual. According to the facial expression that is being displayed, characteristics of an individual’s social nature, such as their credibility, can be assessed [3]. For example, it has been reported that people will invest more money in individuals with trustworthy faces [4]. Thus, financial investment can be used as a task to assess trustworthiness. Oosterhof et al. created a model face that can be altered to depict various facial expressions digitally [5]. Using this model, they investigated facial expressions that elicit trust, and determined that smiling expressions resulted in high trustworthiness, while angry expressions elicited low trustworthiness. On the other hand, in situations where social interaction occurs, elements of social context and linguistic information separate from facial expression, exist as bias. This bias has an effect on social cognition and social behavior. Zaki et al. [6] investigated brain activity when a discrepancy occurs between the non-verbal information of facial expressions and the contextual social language. The results showed that the anterior cingulate gyrus and lateral prefrontal cortex activate when there is a discrepancy between this information. In addition, the results indicated that these areas are active specifically in response to conflicts of social cognition.

In our previous study, we used low resolution brain electromagnetic tomography (LORETA) to investigate the brain activity of individuals receiving stimuli consisting of positive or negative verbal information paired with a smiling face, as well as positive or negative verbal information paired with an expression of disgust. Our results suggest that activity in the parietal lobe increases when observing a smiling face paired with negative verbal information, that is, when the verbal information and facial expression are incongruent [7]. Therefore, when the verbal information and facial expression information were incongruent, activity was demonstrated not only in the anterior cingulate gyrus and the prefrontal area, but also in the parietal lobes, which are also involved in the information interference effect. However, the scope of this previous study was limited to examining the brain activity at the time of observing facial expressions, and it did not include ERP analyses of the process of rating trustworthiness of an individual as measured by the amount of donation.

One past study that used ERP showed that the amplitude of the N170 was enhanced in the occipital-temporal sites in response to seeing a fearful face embedded in a fearful scene [8]. This indicates that the N170 amplitude is affected by emotional context. Another more recent study that used the same experimental paradigm investigated the amplitude of the late positive potential (LPP) that is observed at 300–700 ms after stimulus onset in the parietal cortex, in addition to the N2 component. The results showed that the front-central N2 amplitude was larger in response to an incongruent face-scene combination, such as a face showing fearful emotion in a positive scene, compared to that of a congruent combination such as a face showing fearful emotion in a fearful scene [9]. The N2 is a negative wave that peaks at 200–350 ms after stimulus, and is found primarily over anterior scalp sites [10]. However, the experimental protocols in that study asked the participants to quickly categorize fearful or positive facial expressions, and not to judge the trustworthiness of an individual demonstrating the facial expressions.

Results of recent studies suggest that, when observing a facial expression displaying anger compared to when observing a neutral or happy expression, not only is the early posterior negativity (EPN) (150–300 ms) observed in the posterior area of the head after the stimulus, but the LPP (300–700 ms) in the parietal area is observed as well [11–15]. Previous studies have identified the EPN and LPP as primary components involved in facial recognition processing that are affected by socially affective information. When observing faces that are familiar, such as a romantic partner with a high social relationship or the faces of family, this LPP is strengthened [16–18]. Bublatzky et al. revealed that the LPP appears strongly when a happy expression of an expected partner of future interactions is observed, as when the person whose face was displayed on the screen will be met after the completion of the experiment [19].

The objective of the present study was to determine the change in trustworthiness of an individual when inconsistency in the contextual bias from verbal and facial expression information occurs. In addition, we attempted to clarify the brain wave activity into early components and later components when observing the facial expression of the individual who presented the inconsistency.

## Methods

### Participants

Seventeen healthy students (9 females; mean age, 20.76 years ± 0.56 years) participated in this study. Prior to the experiment, the experimental procedure was explained to the participants. The purpose of the experiment, however, was not explained in order to prevent participant bias. The experimental protocol was approved by the Kio University Ethics Committee. Participants provided written consent to participate in the experiment after receiving an explanation of the procedures involved.

### Stimuli

To present facial stimuli including smiling and angry facial expressions in this experiment, we prepared photos of 12 different individuals (6 females) presenting facial expressions (smiling and angry). Thus, stimuli consisted of 24 facial photos (6 females and 6 males presenting a smiling or an angry expression). The smiling and angry facial expressions that appeared in these photos were created based on the Action Unit Classification of the Facial Action Coding System (FACS) [20].

Verbal stimuli were 66 sentences describing moderately affective events relevant to college students. Sentences were generated by authors M.S. and M.O. Twenty-four volunteers (15 females) provided pilot valence ratings of these sentences using an 11-point Likert scale ranging from 0 (very negative) to 10 (very positive). Based on these ratings, 15 positive and 15 negative sentences were chosen as stimuli for the present study. These verbal cues produced reliable affect-congruent ratings from perceivers (mean rating of positive stimuli, 8.65±0.47; mean rating of negative stimuli, 1.28±0.76). Positive sentences were “I wear nice shirts”, “I alleviated stress”, “I proposed marriage”, “I work as a volunteer”, “I got married”, “I won a game”, “I finished paying back a debt”, “I passed the examination”, “I got a salary raise”, “I received an award”, “I gave someone a helping hand”, “I saved a life”, “I hit the lottery jackpot”, “I had a dream”, and “I made peace with a friend”. Negative sentences were “I said something snide”, “I cybercased”, “I lost my wallet”, “I cheated on a test”, “I broke my watch”, “I ran into debt”, “I failed to enter university”, “I despise my friend”, “I broke my friend’s thing”, “I stole my friend’s thing”, “I pushed my friend over a bridge”, “I ran over my friend”, “I stabbed at my friend with a knife”, “I had a fight with my friend” and “A thief broke into my house”.

### Experimental Conditions

The present task was designed to produce congruence or incongruence conditions in a social setting. Congruence conditions were composed of either a positive sentence × a smile expression (PoSm condition) or a negative sentence × an angry expression (NeAn condition). Incongruence conditions were composed of either a negative sentence × a smile expression (NeSm condition) or a positive sentence × an angry expression (PoAn condition).

### Procedure

**Fig 1:**
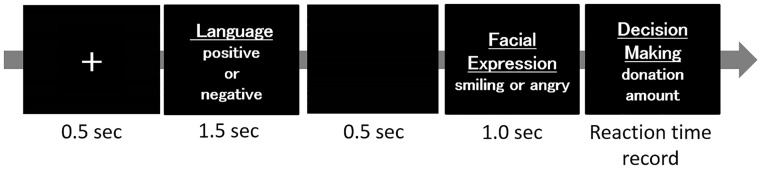
Image presentation and the experimental procedure. Subjects were shown the image of “+” for 0.5 seconds, text for 1.5 seconds, a blank image for 0.5 seconds, a facial expression for 1 second, and, finally, the participants were asked to choose a donation from 0, 2500, 5000, 7500, and 10000 yen. The reaction time was recorded. EEG was recorded for each facial presentation over a total period of 1200 ms starting at 200 ms before a given stimulus (baseline) and ending at 1000 ms after stimulation.

After the electroencephalogram (EEG) sensor net was attached, participants were instructed to watch images on a computer screen while sitting in a chair. In one trial, the screen showed a letter of “+” for 0.5 seconds, a language stimulus for 1.5 seconds, and, finally, the facial expression of a person (960×768 pixel) for 1 second followed by a blank screen (for 0.5 seconds). After being shown the facial expression, participants were asked to choose either 0, 2500, 5000, 7500, or 10000 yen in response to the following question: “If you had 10000 yen on hand, how much money would you give this person if they were having a financial problem?” (Fig 1). Reaction times for the answer were recorded at each trial. A total of 384 trials were administered, with 96 trials for each of the 4 conditions presented randomly. The donation task was formulated based on a previous study [4] demonstrating that people paid more money to individuals who were rated subjectively as having a more trustworthy face. Additionally, in our previous study, we showed that people donated more money to a more trustworthy person [7]. Thus, the present donation task has been shown to be valid to estimate trustworthiness. The combination of prepared facial expression and verbal stimuli were presented randomly. Images were presented on a 17-inch computer screen located approximately 1 meter in front of the participants. These presentations were run in SuperLab (Cedrus Corporation, San Pedro, California, USA) and reaction times were recorded using Response Pads (Cedrus Corporation) and SuperLab (Cedrus Corporation).

### EEG Recording and analysis

EEG recording was performed at 64 sites on the scalp according to the international 10–20 location method (ActiveTwo, BioSemi B.V., Amsterdam, The Netherlands). The sampling frequency was 512 Hz and included low pass filtering at 30 Hz with off-line analyses. Rejection as artifact was performed for amplitudes exceeding 80 μV. Ocular artifacts, such as blinks and large eye movements, were removed from data using a specially designed spatial filter in EMSE Suite 5.4 (Source Signal Imaging, Inc., La Mesa, CA, USA). EEG was referenced to the average reference. A total of 1200 ms of brainwaves were recorded from 200 ms prior to the stimulus (baseline) to 1000 ms after the stimulus for each facial expression presentation.

In order to analyze the cognitive process during viewing facial expressions, the right temporo-occipital EPN and LPP in the centro-parietal area were analyzed. The EPN was deem the earliest component representing personal influence on facial perception [21]. The LPP has been shown to be influenced by emotional and cognitive processes such as attention and reappraisal [21–23]. In the present study, peak amplitudes of the EPN (P8-PO8) within 150–300 ms after the onset of facial expression representation and the mean amplitudes of the LPP (C1-Cz-C2, CP1-CPz-CP2, P1-Pz-P2) within 450–700 ms after the onset of facial expression representation were analyzed. We referred to the methods of Werheid et al. and Wieser et al. in the selection of our time windows [21, 24].

### Statistical Analysis

Because the values for reaction time and amounts of donated money did not have a normal distribution in Shapiro-Wilk tests, two-factorial ANOVA was not used. Reaction time and amounts of donated money were analyzed using the Friedman test across conditions. Wilcoxon signed-rank tests were used for post hoc analyses and the Bonferroni correction was used to adjust the p-values obtained in the post hoc analyses, and the significance level was set at P <0.0083. EPN were analyzed using two-way repeated-measures ANOVA for two binary factors, expression (Smile and Angry) and sentence (Positive and Negative). However, values of the LPP did not have a normal distribution in Shapiro-Wilk tests, and two-factorial ANOVA was not used. The mean amplitude of the LPP for each region was compared between the PoSm and NeSm conditions or between the NeSm and NeAn conditions. A total of three comparisons between each condition were made, and the significance level was set at P <0.0167.

## Results

### Reaction Time and amounts of donated money

**Fig 2:**
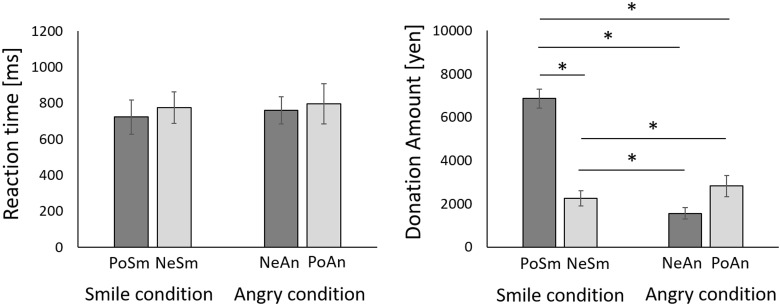
Comparison of reaction times and donations in each condition. Donations for PoSm are significantly greater than those for NeSm conditions. Asterisks in the figure denote significant differences.

According to reaction times, the Friedman test showed a significant main effect (χ2 = 10.059, P = 0.018), but the results of the post hoc test revealed no significant differences between conditions (p>0.0083; PoSm, 723.35±94.54 ms (mean±SE); PoNe, 759.91±76.24 ms; NeAn, 775.29±87.17 ms; PoAn, 795.03±111.51 ms) (Fig 2). Meanwhile, in regards to the amounts of donated money, the Friedman test revealed a significant main effect (χ2 = 35.753, P < 0.001). The amount of donated money in PoSm condition (Mean±SE = 6867.34±437.40 yen) was higher than that in NeSm condition (Mean±SE = 1563.16±270.48 yen), NeAn condition (Mean±SE = 2250.30±350.41 yen) and PoAn condition (Mean±SE = 2823.22±487.98 yen) (p<0.0083). The amount of donated money in PoAn condition was higher than that in NeSm condition (p<0.001). Additionally, the money in NeSm condition was higher than that in NeAn condition (p<0.0083) (Fig 2).

### EPN and LPP using Event Related Potentials (ERP)

**Fig 3:**
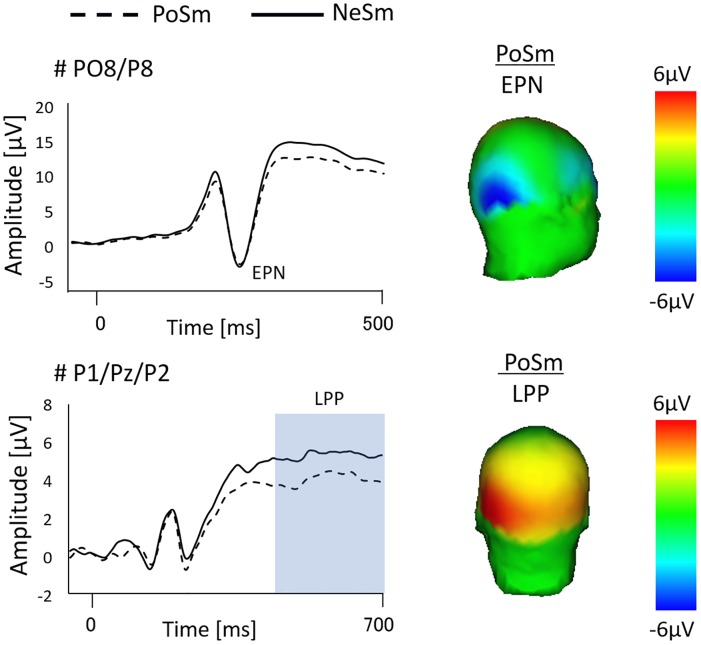
Illustration of the ERP waveform and the topographical map related to the EPN and LPP. ERP waveforms for the right temporo-occipital (PO8/P8) and parietal sensor (P1-Pz-P2) for PoSm (dashed line) and NeSm (solid line) conditions. Topographical maps display the time interval plotted on a backward right (EPN) and back view (LPP).

The ERP-related EPN and LPP at the temporo-occipital and parietal areas in PoSm and NeSm condition are shown in Fig 3. According to the amplitude of the EPN at PO8/P8, statistical analysis using two-factorial ANOVA did not show a significant main effect of expression (F = 0.03, p = 0.85) and sentence (F = 1.75 p = 0.74). There was also no interaction between expression and sentence (F = 0.04, p = 0.825; PoSm, -1.05±0.99 *μ*V; NeSm, -0.51±0.88 *μ*V; NeAn, -1.01±0.99 *μ*V; and NeSm, -0.91±0.97 *μ*V).

**Fig 4:**
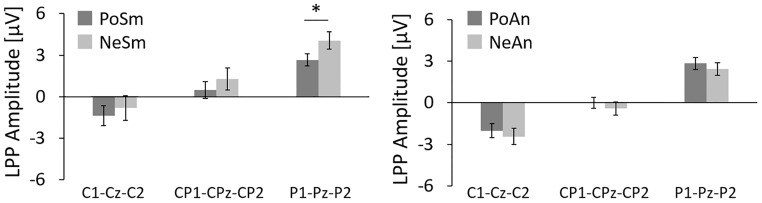
Comparison of mean amplitude of the LPP for each sensor area in the smile expression condition (left) and the angry expression condition (right). There were significant differences at P1-Pz-P2 between the PoSm and NeSm conditions. On the other hand, there were no significant differences between the PoAn and NeAn conditions. Asterisks in the figure denote significant differences.

In regards to the smile expression, according to the amplitude of the LPP at C1-Cz-C2 and CP1-CPz-CP2, there were no significant differences between PoSm (C1-Cz-C2, -1.38±0.72 *μ*V; CP1-CPz-CP2, 0.49±0.61 *μ*V) and NeSm (C1-Cz-C2, -0.80±0.89 *μ*V; CP1-CPz-CP2, 1.28±0.79 *μ*V) (p>0.0167). On the other hand, the amplitude of the LPP at P1-Pz-P2 for NeSm (4.05±0.62 *μ*V) was higher than that for PoSm (2.65±0.43 *μ*V) (p<0.0167). In viewing the angry expression, according to the LPP at C1-Cz-C2, CP1-CPz-CP2, and P1-Pz-P2, there were no differences between NeAn (C1-Cz-C2, -2.02±0.52 *μ*V; CP1-CPz-CP2, -0.004±0.39 *μ*V; P1-Pz-P2, 2.84±0.44 *μ*V) and PoAn conditions (C1-Cz-C2, -2.43±0.58 *μ*V; CP1-CPz-CP2, -0.41±0.49 *μ*V; P1-Pz-P2, 2.43±0.46 *μ*V) (p>0.05) (Fig 4).

## Discussion

This study investigated changes in the perceived trustworthiness of a person showing a facial expression when incongruent information was provided by a sentence followed by a facial expression. In addition, changes in brain wave activity were monitored until a decision of trustworthiness was made. The evaluation of trustworthiness in the present study was determined by the time taken to determine a donation amount and the amount donated. By creating these conditions, we sought to delineate the effects of interactions between preceding verbal information and facial expressions on the perceived trustworthiness of the individual depicting the facial expression.

No significant difference was observed between each condition for the time taken to determine the donation amount. On the other hand, there was a significant increase in the donation amount for the congruent condition, in which a positive sentence was displayed with a smiling facial expression, when compared to the donation amount for the incongruent condition in which a negative sentence was displayed with a smiling facial expression. In fact, the average donation value in the congruence condition was approximately three times higher than that in the incongruence condition.

Krumhuber et al. demonstrated that, for the task of selecting a trustworthy partner, an individual exhibiting a smile will be chosen often [25]. Additionally, if the other individual’s face is a smile, the rate of trust for that person will increase [26]. Thus, smiles are an element that increases trustworthiness. In the present study, when a smiling face was presented with a preceding positive sentence, the donation amount was shown to increase overwhelmingly, demonstrating that trust increased for that individual. Conversely, regardless of whether the facial expression was a smile, if a negative sentence was displayed as the preceding stimulus, the donation amount decreased drastically. On the other hand, when an angry facial expression was shown, regardless of whether the preceding stimulus was a positive or negative sentence, a significant difference was not observed for congruence/incongruence between the sentence and facial expression information, and the donation amount was low for both conditions. These results demonstrate that the donation is not being influenced by the preceding contextual bias. Diéguez-Risco et al. performed a task in which subjects determined if the preceding sentence and the facial expression were congruent or incongruent [27]. The results showed that, while the response time for the congruent condition was faster than that of the incongruent condition when the preceding sentence and the happy facial expression were congruent, the response times for the congruent condition was not faster than that of the incongruent condition when the preceding sentence and the angry facial expression were congruent. These findings demonstrate that angry facial expressions are not influenced significantly by preceding sentences. In our results, when angry facial expressions were displayed as the latter stimulus, a large difference was not observed in the behavior indicator of the donation amount when the preceding sentence was either congruent or incongruent. Our results suggest that a smiling face is affected strongly by the interaction of verbal information and facial expression, as shown by decreased trustworthiness when a smiling facial expression is accompanied by negative verbal information. On the other hand, an angry face is not affected significantly by its preceding sentence, indicating reduced effects of the interaction between the facial expression and verbal information.

We recorded brain wave measurements until the donation decision was made, and a significant difference was not observed for the EPN, while a significant difference was observed in the latter component LPP. Previous studies have shown that the EPN, including the N170 component, acts upon social categorization, such as determining whether the face being observed is the face of an individual the observer knows [28–34] or if there is a gender distinction [35–36]. It is possible that a significant difference between each condition was not observed because those distinctions were not included, as the pictures of faces used in the present study were of individuals unknown to the subjects.

On the other hand, the amplitude of the LPP component is increased compared to neutral stimuli for positive and negative emotional stimulation [37–40]. Further, the amplitude of the LPP component affects higher-order cognitive processes. Breton et al. asked subjects to perform a cognitive task in which they ranked visually the displayed faces of players [41]. They found that, although there was no change in the amplitude of the early N170 component, a change was observed in the amplitude of the LPP. Additionally, when faces are used as the visual stimuli and self-introductions are used as additional auditory stimuli to add meaning to the faces, increases in the LPP are observed in the left and right parietal lobes compared to when just faces are displayed [42]. In our study, we analyzed brain waves of cognitive processes during the decision making process of determining a donation amount for an individual after observing their face, and a significant difference was observed in the amplitude of the LPP component of the left and right parietal lobes. Additionally, the face stimuli used in this study included facial expressions, and, when observing a face with an expression for a long time, differences in the ERP are reflected in the positive components of the late latency range induced in the parietal region [43].

Our results show a significant increase in amplitude of the LPP components observed on both sides of the parietal lobe for the incongruent condition, in which a smile was displayed with a negative sentence, compared to the congruent condition, in which a smile was displayed after a positive sentence. Luo et al. recorded the ERP in response to a gender judgment task where facial expressions were presented visually together with a positive or negative sentence. The study demonstrated that there was a significant increase in the LPP in the parietal lobe when cognitive top-down modulation was evoked by sentences [44]. The parietal LPP, which begins at approximately 400 ms after stimulus onset, is considered to reflect a high-level, cognitive elaboration on motivationally significant stimuli after the stimulus categorization was completed [45]. Therefore, we believe that the increase in the LPP amplitudes in the parietal lobe in response to the current trustworthiness judgment task was also indicative of an increase in cognitive activities.

In conclusion, our results demonstrate that when an individual is presented with a negative expression followed by an image of a smiling face as a positive emotion, an incongruence occurs and the trustworthiness for the individual displaying the smile decreases. In the case of an angry facial expression, however, trustworthiness is the same regardless of a preceding positive or negative expression. In the results of the ERP analysis, the amplitude of the LPP component increased significantly when the preceding negative expression was followed by a smile as a positive emotion, which is an incongruent condition. Together, our results suggest that the LPP amplitude observed from the parietal cortex is involved in the processing of incongruence between verbal and facial expression information.
